# Human Embryos, Induced Pluripotent Stem Cells, and Organoids: Models to Assess the Effects of Environmental Plastic Pollution

**DOI:** 10.3389/fcell.2021.709183

**Published:** 2021-09-03

**Authors:** Dragana Miloradovic, Dragica Pavlovic, Marina Gazdic Jankovic, Sandra Nikolic, Milos Papic, Nevena Milivojevic, Miodrag Stojkovic, Biljana Ljujic

**Affiliations:** ^1^Department of Genetics, Faculty of Medical Sciences, University of Kragujevac, Kragujevac, Serbia; ^2^Department of Dentistry, Faculty of Medical Sciences, University of Kragujevac, Kragujevac, Serbia; ^3^Laboratory for Bioengineering, Department of Science, Institute for Information Technologies, University of Kragujevac, Kragujevac, Serbia; ^4^SPEBO Medical Fertility Hospital, Leskovac, Serbia

**Keywords:** organoids, early development, model disease, environmental pollution, drug screening, bioinformatics

## Abstract

For a long time, animal models were used to mimic human biology and diseases. However, animal models are not an ideal solution due to numerous interspecies differences between humans and animals. New technologies, such as human-induced pluripotent stem cells and three-dimensional (3D) cultures such as organoids, represent promising solutions for replacing, refining, and reducing animal models. The capacity of organoids to differentiate, self-organize, and form specific, complex, biologically suitable structures makes them excellent *in vitro* models of development and disease pathogenesis, as well as drug-screening platforms. Despite significant potential health advantages, further studies and considerable nuances are necessary before their clinical use. This article summarizes the definition of embryoids, gastruloids, and organoids and clarifies their appliance as models for early development, diseases, environmental pollution, drug screening, and bioinformatics.

## Bioinformatics

**FIGURE 1:**
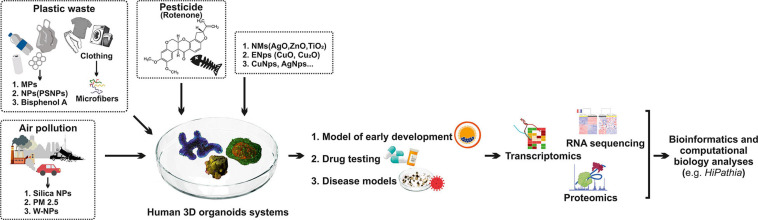
Human 3D organoid systems: a promising platform to study the effect of environmental pollution. Plastic waste, air pollution, pesticides, and various nanomaterials have a negative impact on human health, which is mimicked by organoid systems that serve as a proper model for early development, drug testing, and disease models. As a result of organoids’ exposure to these pollutants, molecular and functional alterations in organoids are noted, so different omic profiling technologies are used to discover these alterations, such as transcriptomic, RNA sequencing, and proteomics. Hence, different bioinformatics tools are used for analyzing omics such as *HiPathia*. MPFs—microplastic fibers; MPs—microplastics; PSNPs—polystyrene nanoplastics; PM 2.5—particulate matter 2.5; PSCs—pluripotent stem cells; W-Nps—tungsten nanoparticles; NMs—nanomaterials; ENps—engineered nanoparticles; Nps—nanoparticles; AgO—silver oxide; TiO_2_—titanium dioxide; ZnO—zinc oxide; CuO—copper oxide.

Various bioinformatics and computational biology analyses are used to evaluate disease model accuracy (Bock et al., 2020; Gendoo, 2020). Different omic profiling technologies are used to discover molecular and functional alterations in organoids (Figure 1). Transcriptomics copy number and structural variation changes (whole-genome sequencing—WGS, whole-exome sequencing—WES), proteomics (protein expression changes), epigenomics, (toxico) genomics, and metabolomics (enrichment of biological pathways) are applied to comprehend or to predict toxicity (Stojkovic et al., 2021b). However, bioinformatics tools are used to analyze omics data (Wu et al., 2018; Mincarelli et al., 2018; Brazovskaja et al., 2019; Bock et al., 2020; Gendoo, 2020; Zink et al., 2020; Figure 1). To elucidate the exact mechanism and understand how various types of pollution particles influence gene changes and signaling pathways in organoids, a reliable method to estimate the activity within pathways is necessary. By estimating the level of activity of stimulus-response sub-pathways (signaling circuits) within signaling pathways, which ultimately trigger cell responses, we can investigate interactions between various environmental and intracellular pollutions to explore the mechanism and origination of human disease, but also prediction of clinical outcomes (Hidalgo et al., 2017; Peña-Chilet et al., 2019; Bojic et al., 2020; Zeng et al., 2021; Wu et al., 2018). A platform like *HiPathia* (Bojic et al., 2020), with vast computational data, enables insight into modeling of various diseases (Figure 1). Results obtained in such a manner may serve as a competent tool for further clinical trials (Hidalgo et al., 2017; Bojic et al., 2020; Falco et al., 2020). *HiPathia* enabled an examination of the effect of carboxyl-modified fluorescent nanosized plastic (polystyrene) items on gene alterations and signaling pathways as reported by Bojic et al. (2020). Moreover, *HiPathia* pointed to several altered circuits, such as the peroxisome proliferator-activated receptor pathway that has a crucial role in lipid metabolism, but also prediction of the clinical outcome which included the *APOC3* circuit, which induces hyperalphalipoproteinemia, thus raising the risk of ischemic cardiovascular disease (Bojic et al., 2020).

RNA massive sequencing (RNA-seq) is the most used technique for gene expression profiling in a single assay (Garrido-Rodriguez et al., 2021). Although it is possible to compare relative gene expression, RNA-seq cannot warrant function at the protein level (Li et al., 2021). Even though throughput is restricted, single-cell RNA-seq identifies infrequent populations of cells with functional significance (Li et al., 2021; Yan et al., 2013). On the other hand, the transcriptome is more significant because it provides information about the specific biological function or gene expression, compared to separate analyses of the genome, the epigenome, and the proteome (Bar and Benvenisty, 2019; Song et al., 2019; Zeng et al., 2021). In the study by Zeng et al. (2021), transcriptome analysis was used to estimate the effect of particulate matter 2.5 (PM2.5) on human embryonic stem cell derived retinal organoids (hEROs) and showed that mitogen-activated protein kinase (MAPK) and phosphoinositide 3-kinase (PI3K)/AKT pathways were involved significantly, while fibroblast growth factors (FGFs), especially FGF8 and FGF10, were decreased, thereby inducing abnormal human retinal development.

In the previously described study (van Dijk et al., 2021; the contribution is a preprint), the RNA-seq analysis has been used to confirm that nylon fibers were less inhibitory for the growth of alveolar organoids (AO) than treatment with component leaching of the polymer or lower numbers of nylon fibers. The Notch1 and Notch2 signaling pathways were downregulated, as well as their ligands Jag1 and Jag2, which are responsible for the development of airway epithelial cells, and club cells (van Dijk et al., 2021; the contribution is a preprint). Winkler and coworkers used human lung organoids to examine the effect of microplastic fibers (MPFs) on organoid growth and their inflammatory effects on the established lung organoids (Winkler et al., 2021; the contribution is a preprint). Quantitative reverse transcription-polymerase chain reaction (qRT-PCR) was used for gene expression analysis of oxidative stress-related genes, lung-specific genes, and inflammatory cytokines (Winkler et al., 2021; the contribution is a preprint). No significant differences in the gene expression of cytokines or oxidative stress-related genes such as superoxide dismutase family genes (*SOD1* and *SOD2*), glutathione detox-related genes [glutathione detox-related genes (*GSTA1* and *GPX1*)], catalase (CAT), and ROS-controlling genes [(NADPH oxidase-2 (*NOX2*), cyclo-oxygenase 1 (*COX1*), NADH dehydrogenase 1 (*ND1*)] were noticed in human lung organoids after exposure to MPFs (Winkler et al., 2021; the contribution is a preprint). The authors also confirmed no significant difference in gene expression responsible for epithelial lung markers such as NK2 homeobox 1 (*NKX2.1*) and Claudin 1 (*CLDN1*) as well as the specific airway lung markers surfactant protein A1 (*SFTPA1*) and surfactant protein C (*SFTPC*) in alveolar-type 2 progenitor cells (AT2 cells), secretoglobin family 1A member 1 (*SCGB1A1*) (club cells), nephrocystin 1 (*NPHP1*), dynein axonemal heavy chain 5 (*DNAH5*) (ciliated cells), and keratin 5 (*KRT5*) (basal cells) in human lung organoids exposed to MPFs and the control group (Winkler et al., 2021; the contribution is a preprint). Schwartz et al. (2015) studied developmental neurotoxicity by using reproducible 3D neural constructs containing vascular and microglial components on synthetic hydrogels after exposure to toxic or nontoxic chemicals. In this study, 3D neural constructs were exposed to a set of 31 control compounds and 39 toxins through day 16 or day 21 and then collected for RNA-seq (Schwartz et al., 2015). RNA-Seq identified differentially expressed genes that included neurogenesis such as GABAergic neurons [e.g., gamma-aminobutyric acid receptor (GABA receptors)], glutamatergic neurons [(e.g., vesicular GABA transporter (*VGAT*) and vesicular glutamate transporters (*VGLUT2*)], cortical neurons [(POU class 3 homeobox 2 (*BRN2/POU3F2*), reelin (*RELN*), BAF chromatin remodeling complex subunit *BCL11B* (*CTIP2*/*BCL11B*)], synaptic markers (e.g., synapsins and synaptic vesicle components), and glial cells [solute carrier family 1 member 3 (*GLAST/SLC1A3*), glial fibrillary acidic protein (GFAP), platelet-derived growth factor receptor alpha (PDGFRA)] in neural constructs in relation to undifferentiated hESCs (Schwartz et al., 2015).

Proteomics is determined as wide-ranging protein analysis enabling recognition, quantification, and posttranslational modification between other related facts in terms of proteins in a cell, tissue, or biofluid (Lindoso et al., 2019). Among other “omics” analysis, proteomics is one of the widely employed methods in bioinformatics and liquid chromatography linked with mass spectrometry and mainly applied in the examination of induced pluripotent stem cells (iPSCs; Walther and Mann, 2010; Lindoso et al., 2019), hiPSC-derived organoids (Hale et al., 2018), or hESC-derived organoids (Nascimento et al., 2019). Using proteomics analysis, Hale et al. (2018) compared organoid-derived glomeruli (OrgGloms) with conditionally immortalized human podocyte cell lines. They elucidated that OrgGloms displayed higher-level matrix extracellular components and an α5(IV) chain of type IV collagen network, while α3 and α4(IV) chains were less expressed. These outcomes emphasize the importance of OrgGloms as a proper 3D model of the human glomerulus in physiological and pathological conditions (Hale et al., 2018). By using shotgun proteomics to examine human cerebral organoids, Nascimento et al. (2019) identified 3,073 proteins associated with various brain developmental stages, especially with neurogenesis, axon guidance, synaptogenesis, and cortical brain development. If there is a need for analyzing alterations in metabolites at the system level, this type of omics is termed metabolomics (Wishart, 2019; Neef et al., 2020; Garrido-Rodriguez et al., 2021).

## Introduction to Blastoids, Embryoids, Gastruloids, and Organoids—The Definitions

Valuable events of early mammalian development and self-organization are demonstrated in various studies on early mouse and human embryos that could be cultured *ex vivo* in the absence of maternal tissues (Deglincerti et al., 2016; Shahbazi et al., 2016). However, early human embryos, including the blastocyst stage, are difficult to obtain, have a small number of cells (<100), and are not easy to physically and genetically manipulate. Therefore, many genetically similar structures should be generated, thus opening possibilities for different analyses, including high-throughput screens and biochemistry-based assays. One of the well-known structures is the blastoid—the first version of a preimplantation blastocyst model made by promotion of the self-organization of mouse embryonic stem cells (ESCs) and trophoblast stem cells (TSCs) (Rivron et al., 2018). The trophoblast and embryonic compartments of blastoids can be physically and genetically modified independently from another blastoid, offering enormous technical advantages compared to blastocysts. Recent studies focused on human blastoids to study and comprehend early human development and prevent pregnancy defects and birth loss (Liu et al., 2021; Yu et al., 2021; Zheng and Fu, 2021). Human blastoids can be generated in a two-step culture process—isolation from human blastocysts or by reprogramming adult human cells (Yu et al., 2021), or in a one-step culture process—by reprogramming skin fibroblasts (Liu et al., 2021). Regardless of the *in vitro* method used for generating human blastoids (one-step or two-step culture), in both cases, it was shown that human blastoids had almost identical size, number of cells, and shape similar to natural blastocysts (Liu et al., 2021; Yu et al., 2021; Zheng and Fu, 2021). In the study of Zheng and Fu (2021), genome-wide expression analysis was used to clearly define molecular similarities of the blastoids with preimplantation human blastocyst. They showed that there are molecular similarities between generated human blastoids and preimplantation human blastocyst. Also, they proved that blastoid cells have crucial characteristics of blastocyst lineages in terms of the ability to generate various stem cell types which are isolated from the blastoids, offering new insights to study early preimplantation and early postimplantation blastocyst development (Zheng and Fu, 2021). However, there are some limitations that need to be reconsidered. For instance, the development of the blastoids is not effective and differs among cell lines from different donors, and between experimental clusters. Also, it was noted that the three lineages in single blastoids developed at different velocities, and their growth in the same dish was not synchronized together with unspecified cell populations with no equivalent in natural human blastocysts (Zheng and Fu, 2021). The other obstacle includes ethical controversies such as the fact that the development of human blastoid *in vitro* is limited in postimplantation stages until 14 days *in vivo* (Zheng and Fu, 2021). The findings of Xiang et al. (2020) might help to improve the ability to culture blastoids up to this limit, by 3D systems for culturing human blastocysts, which effectively promote postimplantation development, although bioethical issues need to be addressed.

Embryoid bodies (EBs) are defined as 3D aggregates of pluripotent stem cells (PSCs) or differentiated cells used as a layout of early development and comprising the three embryonic germ layers (Evans, 2011; Simunovic and Brivanlou, 2017). Additionally, EBs go through the initial development phase similarly to pregastrulating embryos and resemble early teratoma (de Jaime-Soguero et al., 2018). *In vivo* and *in vitro*, cell differentiation depends on morphogen gradients and signals that supply instructive and positional signs (ten Berge et al., 2008; Simunovic and Brivanlou, 2017). For instance, EBs can be used as a model for teratogen-testing platforms, which includes evaluation of chemically induced effects on EB morphology, effects on the differentiation of particular cell types of interest, estimation of the transcriptome or proteome, effects on specific signaling and developmental pathways, and at last, effects upon cellular physiology (Lee S. et al., 2020).

Unlike embryos, which go through defined stages with typical morphologies, such as blastula, gastrula, and neurula stages, embryoid is an artificial construct made from cultured cells that try to imitate all or part of an embryo, or its specific stage (Simunovic and Brivanlou, 2017). The embryoid can be defined as a more organized EB that develops as a result of cell polarization caused by (i) the extracellular matrix (ECM) in the adjacent medium or (ii) the accurate topology of multiple types of cells that represent an embryo at a specified time of development (Simunovic and Brivanlou, 2017).

Unlike embryoids, gastruloids can be defined as an *in vitro* multicellular model of a gastrulating embryo, either in 2D (Etoc et al., 2016) or in a 3D culture system (Turner et al., 2016). Gastruloids are also defined as complex 3D structures with the ability to self-organize *in vitro* and look like developing tissue *in vivo* (Munsie et al., 2017). They are distinct from organoids because they do not essentially recapitulate an organ but rather a developmental process, offering the possibility to create post-implantation models (Munsie et al., 2017) and models to study (Simunovic and Brivanlou, 2017).

For the purposes of this review, we focus primarily on the opportunities and challenges concerning human organoids, and their ability to serve as a model to study the effects of environmental pollution on human health.

## Introduction to Human Organoids

The term “organoids” appeared in the 1950s (Vendrely, 1950) and finally was delineated and systemically elaborated by Eiraku and Sasai (2012). A typical depiction of organoid is explained as a structure in which pluripotent or progenitor stem cells are differentiated into multiple cell populations that self-organize into tissue similar to an organ (Eiraku and Sasai, 2012; Kicheva and Briscoe, 2015; Clevers, 2016) and have the capacity of stable long-term culture and passage (Eiraku and Sasai, 2012). The stem cell sources of the existing cultured organoids are for the most part PSCs—iPSCs, ESCs, and adult stem cells (ASCs). These sources are used to induce various types of organ tissues such as gut (Spence et al., 2011), kidney (Takasato et al., 2015), pancreas (Greggio et al., 2013), brain (Lancaster et al., 2013), retina (Nakano et al., 2012), inner ear (Koehler et al., 2017), lung (Wong et al., 2012), and liver (Takebe et al., 2013). However, the establishment of human AdSC-derived organoids is limited by accessibility to the tissue and prior knowledge of the culture conditions, while an iPSC line, once established from a patient (Brouwer et al., 2016), can be used to repeatedly generate different tissue models without any time limit [that is, beyond the patient’s lifespan (Kim J. et al., 2020; Narsinh et al., 2011)]. Although ASCs can be stimulated to form organoids (Yin et al., 2016), the focus of our review will discuss organoids derived from human iPSCs and ESCs.

## Differences Between 2D and 3D Models

The usage of 2D models is limited compared to superior 3D organoid technology (Ho et al., 2018). The major limitation of 2D culture is cells arranged as a monolayer, providing atypical growth kinetics and cell attachments, therefore not completely presenting the natural microenvironment of the cells (Nicolas et al., 2020). To emulate tissue homeostasis and complex interactions, 3D structures, better known as organoids, are preferably used compared to 2D cultures that are unable to sustain intercell communication (Clevers, 2016). Additionally, 3D systems are rather utilized due to improved cellular membrane integrity, and niche manipulation (Ferraz et al., 2016; McCauley and Wells, 2017; Ho et al., 2018; Chen et al., 2019; Stojkovic et al., 2021b). Also, 3D organoids go through multi-lineage differentiation, creating heterogeneous groups of cells that self-assemble into complex tissue-like structures mimicking physiologically more pertinent microenvironment (Cugola et al., 2016). Many studies show that hESCs and hiPSCs are used for generating 2D and 3D organoids allowing the study of differentiation mechanisms (Pamies et al., 2017; Rosca et al., 2020), the processes involved in embryonic development (Lancaster and Knoblich, 2014; Pamies et al., 2017; Rosca et al., 2020), and the mechanisms involved in various diseases (Lancaster and Knoblich, 2014; Pamies et al., 2017; Rosca et al., 2020; Stojkovic et al., 2021a), drug testing (Lancaster and Knoblich, 2014), and toxicity connected with environmental pollutants (Truskey, 2018; Rosca et al., 2020; Stojkovic et al., 2021b). However, it is well known that organoids have tremendous potential in drug screening and personalized medicine (Kondo and Inoue, 2019; Kim J. et al., 2020). By discovering organoids, their use made the drug development faster, more effective, and ethically more justified than using animal models for the same purpose (Aboulkheyr Es et al., 2018). Different new medical treatments that were developed for the human disease often manifest many limitations (e.g., problems with predictions of outcomes, time-consuming drug testing, or differences relating to the patient as an individual) (Eglen and Randle, 2015; Dedhia et al., 2016). Therefore, organoid culture based on a particular individual or disease will advance into the potential instrument for adequate therapy (Lehmann et al., 2019).

Regarding the use of organoids as a suitable 3D model, certain obstacles need to be overcome. (1) There is lack of adequate cellular microenvironment, such as endothelial or immune cells (Koike et al., 2019); this problem can be solved by coculturing additional missing cells with organoids. (2) Prices for organoid establishment are relatively high compared to traditional cell lines (although organoids cost less than mouse or fish models). (3) 3D models mirror only specific organ tissue, not the whole organism, and therefore lack interorgan communication. However, progressive endeavors to solve this problem emerge. For instance, a few organoids have been joined with the aim to examine communication between the liver, pancreas, and gastrointestinal tract (Adhya et al., 2018), or to study the interplay between the brain and hormone-producing organs (Xiang et al., 2020). (4) There are no widely accepted protocols standardized for organoid establishment. (5) Heterogeneity in terms of variation between individuals and protocols results in different outcomes (Truskey, 2018; Kim J. et al., 2020). To overcome this obstacle, single-cell profiling technologies for transcriptome and epigenome analysis might be crucial regarding highly correct assays appropriate for this purpose (Kim J. et al., 2020). It should be emphasized that the advantages of using organoids are much greater compared to their disadvantages—of course, both should be taken into account.

## Organoids as Models of Early Development

Besides the improved research of signaling pathways in cell specification and organogenesis, organoids illustrate the physical basis of tissue and organ forming (Lancaster and Knoblich, 2014). Primary sources of organoids are ESCs, iPSCs, and fetal tissues (Spence et al., 2011; Nakano et al., 2012; Wong et al., 2012; Greggio et al., 2013; Lancaster et al., 2013; Takebe et al., 2013; Takasato et al., 2015). The development of tissues such as the stomach, brain, and pancreas has been studied through gradual differentiation of iPSCs and ESCs to organoids by adjusting signaling pathways such as Wingless-related integration site (WNT), bone morphogenetic protein (BMP), and FGF (Greggio et al., 2013; Lancaster et al., 2013; McCracken et al., 2011). Fetal pulmonary organoids are being utilized to demonstrate the signaling interaction between exogenous FGFs (essential for endothelial network assembly) and the vascular endothelial growth factor A (VEGF-A) pathway known to suppress the forming of the endothelial network and the cross talk with the Sonic hedgehog (SHH) pathway that promotes epithelial and endothelial morphogenesis (Mondrinos et al., 2014). When it comes to pancreas development, the loss of F-box and WD repeat domain-containing 7 (Fbw7) in CK19 cytokeratin 19 (Krt19)^+^ adult pancreatic ductal cells *in vivo* led to stabilization of the transcription factor Neurog3 (*Ngn3*), resulting in reprogramming of ductal cells to insulin-secreting beta cells (Sancho et al., 2014). Intestinal organoids–enteroids resulted in structures having leucine-rich repeat-containing G-protein-coupled receptor 5 (Lgr5^+^) intestinal SCs and other differentiated cells localized equally to the *in vivo* organization (Cao et al., 2015). The forebrain cerebral cortex was also developed using novel protocols for 3D brain-like tissue development (Lancaster and Knoblich, 2014; Shahbazi et al., 2016), therefore confirming the vast potential of brain organoid research. In 2017, Koehler et al. (2017) reported the derivation of inner ear organoids using human PSCs and modulating FGF, BMP, TGF-β, and WNT signaling, generating organoids with sensory epithelia that are innervated by sensory neurons. This method significantly promoted further studies of human inner ear development and research on regenerative or drug therapies for hearing loss.

## Organoids in Drug Screening

Many studies showed how healthy organoids can be used in the assessment of drug toxicity such as cardiotoxicity (Eder et al., 2016), nephrotoxicity (Takasato et al., 2015), and hepatotoxicity (Kostadinova et al., 2013; Katsuda et al., 2017). On the other hand, organoids are used to study the effect of some drugs on preexisting diseases, such as primary tumors (Jabs et al., 2017; Abbasi, 2018; Vlachogiannis et al., 2018), rare genetic diseases including cystic fibrosis (Fleischer et al., 2020), neurological diseases (Lee S. E. et al., 2020; Costamagna et al., 2021), and infectious diseases (Zhou et al., 2017; Heo et al., 2018). Other studies reported the use of cancer organoid lines, for example colorectal cancer (CRC) organoid lines to screen 83 drugs (van de Wetering et al., 2015), breast cancer organoid lines for testing inhibitors human epidermal growth factor receptor (HER) signaling pathway (Reid et al., 2018), or bladder cancer organoid lines for testing 26 drugs (Lee et al., 2018). Also, organoid technology aims to supply functional biological structures that can be transplanted into patients in the near future, although precise characterization and validation of organoids as accurate models of human biology are required (Bock et al., 2020). AOs and proximal airway air–liquid interface cell culture systems are advantageous for the examination of antiviral compounds against severe acute respiratory syndrome coronavirus clade 2 (SARS-CoV-2). The next drug for the treatment of COVID-19 has been examined heretofore: IFN type I, IFN type III, remdesivir, camostat mesylate [a cofactor transmembrane protease serine 2 (TMPRSS2) inhibitor], E-64d (an inhibitor of the endosomal cysteine proteases cathepsin B and L), and a library of FDA-approved drugs (the Prestwick collection) (Han et al., 2021; Huang et al., 2020; Lamers et al., 2021). Both remdesivir and camostat mesylate showed antiviral capacity and decreased SARS-CoV-2 N levels (Huang et al., 2020). Imatinib, mycophenolic acid, and quinacrine dihydrochloride diminished SARS-CoV-2 infection of hPSC-derived lung organoids (Han et al., 2021). Pretreatment with IFN-λ1 abolished viral replication in bronchioalveolar organoids. Angiotensin-converting enzyme 2 (ACE2) expression is regulated by androgen signaling and represents an important risk factor of adverse COVID-19 outcome in male adults. This is confirmed by antiandrogenic drugs, which diminished ACE2 expression and prevented SARS-CoV-2 infection of human hESC-derived lung organoids (Samuel et al., 2020).

## Embryos, Their “Surrogates” and Organoids as a Model for Environmental Pollution

The systematic assessment of the global effects of environmental pollution on human health has become increasingly quantitative in the last decades. Humans and animals are constantly exposed to many environmental pollutants and stressors—at least those associated with air pollutants, modern chemicals in the home, food, and beverages/water (Briggs, 2003; Manisalidis et al., 2020). Despite the properly planned high-throughput screening that tried to illuminate the model of action of pollutants (Kavlock et al., 2012), further examinations are necessary to completely understand the exact mechanism by which pollutants cause pathology (Kavlock et al., 2012). There is a wide range of environmental pollutants (Table 1). Due to their omnipotent presence and extensive usage in every aspect of the industry, plastics have become one of the most severe environmental pollutants (Bouwmeester et al., 2015). Plastics in the environment have two forms depending on the size, microplastics (MPs, diameter <1 mm), and nanoplastics (NPs, diameter <100 nm), and can be found in water, ground, food, and various objects and materials (Bouwmeester et al., 2015; Prata, 2018; Toussaint et al., 2019). Hence, polystyrene, as one of the most utilized sorts of plastic, especially in packing food and drinks, construction, computer printers, and other industries, requires further research (Gu et al., 2019; Li-Juan et al., 2019). A detailed study on the possible effects of polystyrene NPs (PSNPs) on the transcription profile of preimplantation human embryos and hiPSCs has recently been, for the first time, conducted by our group (Bojic et al., 2020). Applying the gene set enrichment analysis and *HiPathia*, this study showed that PSNPs led to downregulation of *LEFTY1* and *LEFTY2*, pluripotency genes, and upregulation of *CA4* and *OCLM*, genes related to eye development. Also, there was a significant impact of PSNPs on genes responsible for the development of atrioventricular valve and cellular components. The RNA-seq analysis showed that PSNP intracellular pollution might cause different clinical outcomes, including abnormal early development and several detrimental diseases (Bojic et al., 2020). MPs are omnipresent in the environment (Prata et al., 2020) and are continuously released into the atmosphere. Most MPs consist of MPFs coming from synthetic clothing, fabric, and upholstery (Henry et al., 2019), but mostly from polyester (Dris et al., 2017). One study examined the effect of MPFs on lung organoids derived from tissue-resident ASCs of healthy donors. The organoids were exposed to various MPF concentrations (1, 10, and 50 mg L^– 1^) and analyzed by optical microscopy, scanning electron microscopy (SEM), and confocal microscopy. Gene expression assessment of lung-specific genes, inflammatory cytokines, and oxidative stress-related genes was performed by qRT-PCR and showed no significant differences when compared to the control group (Winkler et al., 2021; the contribution is a preprint). Even though MPFs did not have an adverse effect on lung organoids, there was the polarization of the cell growth along the fibers, similarly to organoid-covered plastic fibers with a cellular layer in the study of van Dijk et al. (2021; the contribution is a preprint). Such outcomes implied possible negative effects of MPFs. Hence, a recent study by van Dijk et al. (2021) showed that the growth of murine and human lung organoids was inhibited 14 days after exposure to nylon microfibers. This was confirmed by light and fluorescence microscopy. However, the effect of polyester on human organoid growth was less profound. In the same study, it has been proved that nylon microplastics did not affect fully develop 14-day organoids, suggesting that nylon microplastics have a huge impact on developing organoids (van Dijk et al., 2021; the contribution is a preprint). This is explained by the negative impact of nylon microplastics on the top five enriched pathways for downregulated and upregulated genes crucial for epithelial development and function. Even though van Dijk et al. (2021) supposed that bisphenol A is the main reason for lung organoid growth inhibition, incubation of lung organoids with bisphenol-A did not affect organoid growth. Eventually, this study suggested that nylon microplastics can negatively affect children and people with chronic or seasonal respiratory diseases. However, very few studies show the detrimental effect of plastic waste on an individual’s development and metabolism. Bisphenol A, also known as the most examined endocrine disruptor (Corrales et al., 2015), is a widely used chemical that can be found almost everywhere—soft plastic bottles, the lining of aluminum food cans—and can harm metabolic and reproductive function (Choi et al., 2016). Choi et al. (2016) examined the effect of acute exposures to bisphenol A on bovine embryo development *in vitro* at environmentally proper concentrations (1 and 10 ng mL^– 1^) at 3.5–7.5 days post-fertilization. They showed that blastocyst development was impaired, embryo quality was decreased, and glucose utilization was increased, although cell number was not altered after exposure to 10 ng mL^– 1^ bisphenol A (Choi et al., 2016). Some studies displayed the effect of low-dose bisphenol A on the early differentiation of hESCs into mammary epithelial cells in 3D conditions (Yang et al., 2013). Another study showed that a low dose of bisphenol A negatively affected hESCs’ differentiation into prostate organoids (Calderon-Gierszal and Prins, 2015). These two studies addressed the toxic effects of bisphenol A on the reproductive systems using hESCs differentiated into mammary epithelial cells and human prostate organoids in 3D conditions (Yang et al., 2013; Calderon-Gierszal and Prins, 2015).

**TABLE 1:** Various environmental pollutants and their employment.

Name of pollutant	Employment
Plastics	
PSNPs	Packing food and drinks, construction, computer industry, etc.
MPFs	Synthetic clothing, fabric, and upholstery
Nylon microfibers	Tires, synthetic clothing, tennis balls, laundry and dishwasher, pods/tablets, cigarette, butts, glitter, wet wipes, tea bags
Bisphenol A	Soft plastic bottles, the lining of aluminum, food cans
Silica Nps	Industry of glass, foundries, construction, ceramics and chemical, plastics, rubber, water filtration, and agriculture
PM2.5	Emitted during the combustion of solid and liquid fuels, such as for power generation, domestic heating and in vehicle engines
W-Nps	Nanotechnology, metallurgy and fusion technology,
Rotenone	Pesticide, used in lakes and reservoirs to kill fish
Pharmaceutical drugs, pesticides, flame retardants, PAHs, lead, mercury, acrylamide, bisphenol, deltamethrin, triphenyl phosphate, methyl mercuric(II) chloride, saccharin, methyl mercury, berberine chloride, saccharin, D-glucitol, acetaminophen, acetylsalicylic acid, and L-ascorbic acid	Wide industrial and pharmaceutical usage
Lead, mercury, glyphosate, thallium	Drinking water, food, or the earth
NMs	
AgO, ZnO, TiO_2_, MWCNT	High-strength composites, energy storage and energy conversion devices, sensors, field emission displays and radiation sources, hydrogen storage media and heterogeneous catalysis, photocatalytic wastewater treatment and hydrogen production, solar cells and gas sensing
TiO_2_, ZnO, CeO_2_ crystalline silica DQ12	Wastewater treatment and hydrogen production, solar cells and gas sensing
CuO, Cu2O- (PVP) Nps	Industrial processes (e.g., catalyst), in commercial products (e.g. sunscreen), as anti-microbial agents
AgNps	Medical, food, health care, consumer, industrial purposes
CdTe, CuO Nps	Solar cells, IR detectors, radiation detectors, electrooptic modulators, industrial processes

*Polystyrene nanoplastics NPs, PSNPs; microplastic fibers, MPFs; cadmium telluride, CdTe; particulate matter 2.5, PM2.5; tungsten nanoparticles, W-Nps; polycyclic aromatic hydrocarbons, PAHs; nanomaterials, NMs; nanoparticles, Nps; silver oxide, AgO; zinc oxide, ZnO; titanium dioxide, TiO_2_; multiwalled carbon nanotubes, MWCNT.*

Besides the plastics, there are numerous environmental pollutants whose effect was examined on organoids (Table 2). The respiratory tract is the first target of numerous professional noxas, such as silica, especially present in industries of glass, foundries, construction, ceramics, chemical, plastics, rubber, water filtration, and agriculture. Di Cristo et al. (2020) confirmed the suitability of the 3D airway model regarding the simulation of working conditions of people exposed to silica nanoparticles (SiO_2_ Nps). A 3D mucociliary tissue model of the primary human bronchial epithelium was exposed to SiO_2_ Nps for 12 weeks; the viability of the 3D airway model was assessed by AlamarBlue (resazurin) assay, whereas the integrity of the tissue was measured by transepithelial electrical resistance (TEER) and assessment of the membrane proteins’ expression was performed by Western blot analysis. Interestingly, no adverse effect of SiO_2_ Np exposition *in vitro* was confirmed, suggesting the effectiveness of the 3D airway model regarding mucociliary system clearance and respiratory defense mechanisms after SiO_2_ Np exposition (Di Cristo et al., 2020). When it comes to inhalation pollutants, besides silica, particulate matter 2.5 (PM2.5), an air pollutant of very small size (≤2.5 μm), is present mostly in car gas emission and is related to numerous lung pathologies, such as asthma, COPD, or lung cancer (Li et al., 2018). For that reason, one study examined whether organic PM2.5 extract caused the same cytotoxic effect in *in vitro* and *in vivo* conditions (Ferraz et al., 2016). Results displayed that PM2.5 had a cytotoxic effect on A549 cells cultured in a monolayer or 3D, by reducing mitochondrial dehydrogenase activity and cell membrane integrity, respectively (Ferraz et al., 2016). However, the exact mechanism by which PM2.5 influences lung development and leads to various lung pathologies remains unclear. Thereupon, a study was conducted in order to decipher the developmental toxicity of fine diesel PM (dPM2.5) exposure during hPSC-derived alveolar epithelial cell (AEC) differentiation and 3D multicellular AO development (Kim J. H. et al., 2020). Results showed that dPM2.5 harmed the AEC differentiation and led to upregulation of NADP oxidases and inflammation. Also, exposition to PM2.5 caused epithelial-to-mesenchymal transition during AEC and AO development. Remarkably, for the first time, there was an upregulation of two important molecules—ACE-2 and TMRPSS2—in both hPSC-AECs and AOs treated with dPM2.5 (Kim J. H. et al., 2020). Importantly, ACE-2 is a protein that enables the entry point for the coronavirus to invade and infect a wide range of human cells and causes the SARS-CoV-2 (Hoffmann et al., 2020). This study displayed the alveolar development toxicity and the rise of SARS-CoV-2 permissiveness of PM2.5 exposed cells, making this hPSC-based 2D and 3D alveolar induction model beneficial in terms of environmental toxicity and SARS-CoV-2 virus examination (Hoffmann et al., 2020). Not just lung organoids, but also retina, turned out as suitable for PM2.5 toxicity research, which was confirmed by Zeng et al. (2021). They examined the effect of PM2.5 on the development of the human retina by using hEROs. In this study, it was shown that the development of hEROs was influenced by PM2.5 exposure in a dose-dependent manner (25, 50, and 100 μg/mL), by reducing cell proliferation and supporting cell apoptosis, which resulted in abnormal human retinal development (Zeng et al., 2021). Finally, to encircle a wide range of lung organoid employment, it should be noted that the effect of tungsten nanoparticles (W-NPs), utilized in nanotechnology, metallurgy, and fusion technology, can be also successfully examined on organoids. The International Thermonuclear Experimental Reactor (ITER) is a project that examined potential effects of W-Nps that could be emitted in air and environment and subsequently affect the respiratory tract by inhalation (George et al., 2019). The latest study examined the acute toxicity of W-Nps on MucilAir^TM^, a 3D *in vitro* model of the human airway epithelium. W-Nps had a restricted influence in terms of toxic effects, cellular absorption, and W transfer over the lung epithelium leading to a decrease in barrier integrity, whereas there was no effect on metabolic activity or cell viability, except a transient increase in IL-8 secretion (George et al., 2019). This research might offer initial data about biokinetic lung models for ITER-like tritiated W-Nps. All of these outcomes can be utilized to settle novel safety policies and radiation protection modes. Besides lungs, certain toxicants may affect the neurological system. For instance, rotenone, a well-deciphered, widely utilized pesticide to control fish populations, is proved to be neurotoxic and leads to Parkinson’s disease (Richardson et al., 2019). To examine that in more detail, the immortalized cell und human mesencephalic (LUHMES) cells were used to study cellular toxicity, resurgence, and adaptability subsequently to rotenone exposition (Harris et al., 2018). 3D LUHMES was exposed to rotenone (100 nM, 24 h) which led to a decrease in complex I activity, ATP, mitochondrial diameter, neurite outgrowth, and transcriptomic changes. Subsequently to compound removal, all of these adverse effects were overcome, due to cells’ adaptation to short-term rotenone exposure. In order to test resilience, cells were reexposed to rotenone after the washout and recovery period. There were transcriptomic alterations in genes, such as nuclear factor erythroid-derived 2-like 2 (*NEF2L2*) which regulates the response to oxidative stress, activating transcription factor 4 (*ATF4*) branch of the unfolded protein response activated in response to endoplasmic reticulum (ER) disturbances or proteotoxicity, and excitatory amino-acid carrier 1 (*EAAC1*), a high-affinity Na+-dependent L-glutamate/D,L-aspartate cell-membrane transport protein that were downregulated on day 14 but unaltered in pre-exposed aggregates. Dopamine active transporter (*DAT*) and Caspase 3 (*CASP3*) were only changed after reexposure to rotenone, while thymidylate synthetase (*TYMS*) and centromere protein U (*MLF1IP*) were downregulated in both single-exposed and pre-exposed aggregates. This study enables insight into the effect of rotenone in neurodegenerative diseases and displayed 3D systems as an excellent tool for neurotoxicity research. Another detailed study examined the neurotoxic profile of 87 compounds (Table 1) widely utilized in various industrial sectors (Sirenko et al., 2019). All of the compounds were tested using hiPSC-based 3D neural cultures. Calcium oscillations—which are related to necrosis or disease progression—are detected in 57% of the analyzed compounds (Sirenko et al., 2019). Characterization of oscillation profiles in 3D neural cultures was performed through multi-parametric analysis while cellular and mitochondrial toxicity was estimated by high-content imaging. This model turned out as beneficial and illuminating toward the exact neurological effect of such a huge complex of compounds. Besides brain and lung organoids, various compounds (Table 1) have been examined on other types of 3D systems—liver, heart, intestine, skin, placenta, and retina (Table 2). The liver, as the metabolic center, represents a perfect target for toxicology research. A study by Forsythe et al. (2018) examined the dose-response toxicity of lead, mercury, glyphosate, and thallium on the liver and cardiac organoids within 48 h. The effects of all compounds were estimated by cytotoxicity and viability staining, ATP activity evaluation, IC50 value, and cardiac beat activity. Likewise, it turned out that all tested compounds have a toxic effect on both liver and cardiac organoids, especially thallium (Forsythe et al., 2018). Not just heavy metals, but also nanomaterials (NMs), due to ubiquitous employment and constant public exposure rose concerns regarding the exact impact of NMs on human health. Therefore, one study examined effects on the liver organoids of single and numerous exposures of NMs [silver oxide (AgO), zinc oxide (ZnO), titanium dioxide (TiO_2_), and multi-walled carbon nanotubes (MWCNT)] (Kermanizadeh et al., 2014). Results showed that numerous exposures were significantly more harmful, specifically AgO and ZnO. The cytotoxic effect was analyzed by the level of cytotoxicity, cytokine secretion, lipid peroxidation, and genotoxicity. Later on, the same group (Kermanizadeh et al., 2019) examined a 3D human liver microtissue (MT) repeatedly exposed to minimal NM concentrations, including TiO_2_, ZnO, CeO_2_, and crystalline silica DQ12. NM cell toxicity effect was observed by analysis of cell membrane integrity and aspartate aminotransferase (AST) activity, pro/anti-inflammatory response, and hepatic function. NM-treated MT displayed a concentration-dependent penetration of NMs profoundly within the tissue, while AST assessment turned out to be unsuitable in this experiment, whereas the cytokine analysis (IL6, IL8, IL10, and TNF-α) proved useful in highlighting recovery periods. Overall, this study emphasized the great advantage of liver MT in nanotoxicology research and highlighted the nanotoxicological assessment on liver MT beyond 2 weeks as unsuitable, due to the aging effect on cells. Since ingestion is a possible route of environmental toxin intake, the gastrointestinal (GI) tract is inevitable for environmental pollution research. Even though toxicological studies on engineered nanoparticles’ (ENps) influence on the GI tract are minimal (McCauley and Wells, 2017), still, there are promising outcomes that could reveal the impact of nanoparticles on the gut. Henson et al. (2019) examined the cytotoxicity of cupric (II) oxide (CuO) and Cu_2_O-polyvinylpyrrolidone (PVP)-coated Nps and copper ions on rat intestine epithelial cells (IEC-6) and human intestinal cells, 2D and 3D models. The mechanism by which copper nanoparticles cause toxicological properties includes reactive oxygen species (ROS) forming, reduction of cellular glutathione, mitochondrial membrane depolarization (Thit et al., 2015; Wang et al., 2021), mitochondrial membrane damage (Wang Y et al., 2012; Wang Z et al., 2012), and the release of toxic Cu ions (Fröhlich, 2013). In line with these outcomes, Henson et al. (2019) estimated cell viability by MTT assay, H_2_O_2_, and glutathione (GSH) detection, and mitochondrial membrane potential. The CuO Nps were more cytotoxic to the rat 2D intestinal model than the human 3D model, probably due to differences between 2D and 3D cultures themselves, and/or differences between species origins (rats vs. humans). Finally, CuO Nps were cytotoxic to rat and human intestinal cells in a dose- and time-dependent manner, proposing therefore that Cu_2_O-PVP Nps are toxic due to their dissolution to Cu ions, while CuO Nps have innate cytotoxicity, without dissolving to form Cu ions. Another detailed research observed the impact of AgO, CuO, ZnO, and TiO_2_ Nps on the EpiIntestinal tissues (Markus et al., 2021). Outcomes again displayed a decline in viability and tissue barrier debilitation after exposition to CuO, ZnO, and SWCNT Nps. Additionally, in culture supernatants 24 h after incubation, there was the dosage-dependent release of IL-8 (inflammatory response) for CuO and ZnO, together with 8-isoprostane release (oxidative stress) for CuO. However, Ag Nps had no side effects on the intestinal microtissues *in vitro*, as it was displayed earlier (Burduşel et al., 2018). Also, AgNp toxicity effect was examined on a 3D epidermal model and a 2D keratinocyte model (Chen et al., 2019; Crosera et al., 2009). *In vitro* examination displayed that a similar dosage of AgNps emerged in considerable oxidative damage and inflammation-related cytotoxicity. However, only 2D keratinocyte cultures were affected by Ag Np toxicity (Chen et al., 2019), which can be explained by the abovementioned drawbacks of monolayer culture when compared to 3D systems. This distinction regarding the different toxicological effects on 2D and 3D cultures was also confirmed in placental toxicity research. Accordingly, a recent study utilized scaffold-free hanging drop technology and a 3D coculture MT model similar to *in vivo* placental tissue (Muoth et al., 2016). Outcomes from this study displayed that secretion levels of human chorionic gonadotropin (hCG) were notably more elevated in 3D when compared to 2D cell cultures (Muoth et al., 2016). Also, it was displayed that cadmium telluride (CdTe) and CuO Nps negatively affected MT viability and hCG secretion (Muoth et al., 2016).

**TABLE 2:** Toxicological effects of environmental pollutants on various organoids.

Name of pollutant	Cell type	Toxicological effect	References
PSNPs	Preimplantation human embryos and hiPSCs	-Downregulation of *LEFTY1* and *LEFTY2* -Upregulation of *CA4* and *OCLM* -Impact on genes responsible for development of atrioventricular valve and cellular components	Bojic et al., 2020
MPFs	Lung organoids	-Polarization of the cell growth along the fibers	Winkler et al., 2021
Nylon microfibers	Murine and human lung organoids	-Upregulation and downregulation of more than 500 genes crucial for epithelial development and function -Developing organoid growth inhibition	van Dijk et al., 2021
Bisphenol A	hESC-derived mammary epithelial cells	-Inhibition of differentiation of hESC into mammary epithelial cells	Yang et al., 2013
	hESC-derived prostate organoids	-Inhibition of differentiation of hESC into prostate organoids	Calderon-Gierszal and Prins, 2015
Silica Np	3D mucociliary tissue model of primary human bronchial epithelium	-No adverse effect	Di Cristo et al., 2020
PM2.5	A549 cells cultured in a monolayer or 3D	-Mitochondrial dehydrogenase activity reduction -Cell membrane integrity reduction	Ferraz et al., 2016
	hPSC-derived AEC and 3D multicellular AO	-Impairment of the AEC differentiation -Upregulation of NADP oxidases -Upregulation of pro-inflammatory IL-6 -Epithelial-to-mesenchymal transition during AEC and AO development -Upregulation of ACE-2 and TMRPSS2	Kim J. H. et al., 2020
	hESC-derived retinal organoids (hEROs)	-Reduction of cell proliferation -Cell apoptosis promotion	Zeng et al., 2021
W-Nps	MucilAir^TM^-3D *in vitro* model of the human airway epithelium	-Slight decrease in barrier integrity -Transient increase in IL-8 secretion	George et al., 2019
Rotenone	Immortalized cell Lund human mesencephalic (LUHMES) cells	-Downregulation of *NEF2L2*, *ATF4*, *EAAC1*, *TYMS*, and *MLF1IP* genes	Harris et al., 2018
Pharmaceutical drugs, pesticides, flame retardants, PAHs, lead, mercury, acrylamide, bisphenol, deltamethrin, triphenyl phosphate, methyl mercuric(II) chloride, saccharin, methyl mercury, berberine chloride, saccharin, D-glucitol, acetaminophen, acetylsalicylic acid and L-ascorbic acid	hiPSC-based 3D neural cultures	-Calcium oscillations	Sirenko et al., 2019
Lead, mercury, glyphosate, thallium	Liver and cardiac organoids	-Integrity and viability reduction -Decrease in ATP activity -Depressive effects on heart beat activity	Forsythe et al., 2018
AgO, ZnO, TiO2, MWCNT	3D human liver MT	-Concentration-dependent decrease in cell membrane integrity Concentration-dependent increase in IL8 and IL10, -Higher levels of TBARS -Increase in DNA strand breaks	Kermanizadeh et al., 2014
TiO_2_, ZnO, CeO_2_ crystalline silica DQ12	3D human liver MT	-Reduction of albumin production -Alterations in cytokine production levels (TNF-α, IL-6, IL-8, and IL-10) -NM penetration deep into the MT	Kermanizadeh et al., 2019
CuO, Cu2O (PVP) Nps	(IEC-6)-rat small intestine epithelial cells, EpiIntestinal^TM^ (SMI-100)-3D model of the human small intestine	-Decrease in cell viability -Decrease in cellular GSH -Increase in H_2_O_2_ -Mitochondrial membrane depolarization	Henson et al., 2019
AgO, CuO, ZnO, TiO2, SWCNT Nps	EpiIntestinal tissues -3D model of the human small intestine	-Dose-dependent reduction of the tissue barrier and viability -Dose-dependent release of IL-8 for CuO and ZnO -Dose-dependent release of 8-isoprostane for CuO	Markus et al., 2021
AgNps	3D epidermal model-EpiKutis	-No adverse effects	Chen et al., 2019
	2D keratinocytes	-Increased levels of ROS, MDA, IL-1α, IL-6, IL-8 -Cell viability and membrane permeability decrease	
CdTe, CuO Nps	3D coculture microtissue (MT) model of a human placenta	-Decrease in MT viability -Reduction of hCG release	Muoth et al., 2016

*Nanomaterials, NMs; nanoparticles, Nps; polystyrene NPs, PSNPs; human-induced pluripotent stem cells, hiPSCs; microplastic fibers, MPFs; human embryonic stem cells, hESCs; particulate matter 2.5, PM2.5; alveolar epithelial cell, AEC; alveolar organoid, AO; nicotinamide adenine dinucleotide phosphate, NADP; hESC-derived retinal organoids, hEROs; tungsten nanoparticles, W-Nps; angiotensin-converting enzyme 2, ACE-2; cofactor transmembrane protease serine 2, TMRPSS2; polycyclic aromatic hydrocarbons, PAHs; silver oxide, AgO; zinc oxide, ZnO; titanium dioxide, TiO_2_; multiwalled carbon nanotubes, MWCNT; thiobarbituric acid reactive substances, TBARS; reactive oxygen species, ROS; glutathione, GSH; hydrogen peroxide, H_2_O_2_; malondialdehyde, MDA, Cupric (II) oxide, CuO; Cu_2_O-polyvinylpyrrolidone, PVP; rat small intestine epithelial cells, IEC-6; 3D model of the human small intestine, SMI-100; cadmium telluride, CdTe; copper oxide, CuO; microtissue, MT; human chorionic gonadotropin, hCG.*

## Organoids as Disease Models

There have been numerous studies utilizing organoid cultures to research congenital and acquired human diseases (McGuigan and Sefton, 2006; Spence et al., 2011; Dekkers et al., 2013; Lancaster et al., 2013; Schwank et al., 2013; Zhou et al., 2017; Praharaj et al., 2018). Here, the focus will be only on infectious disease, coronavirus precisely, since the current pandemic situation in regard to SARS-CoV-2 infection requires rapid and thorough observations. In such a manner, the accent of this section will be focused on lung organoids in the model of SARS-CoV-2 infection highlighting, again, a wide range of employment of organoids in research of almost every known pathology. Respectively, the susceptibility to SARS-CoV-2 and its impact on the human lung AECs were examined in different *in vitro* models. In these studies AOs were derived from various cell types: primary small airway basal cells (Lamers et al., 2020), single adult human alveolar epithelial type II or KRT5+ basal cells (Katsura et al., 2020; Salahudeen et al., 2020), multipotent SOX2+SOX9+ lung bud tip progenitor cells, and either HESCs or iPSCs (Huang et al., 2020). In all of these studies, alveolar epithelial type II-like cells were cultured in 3D as monolayered epithelial spheres or as 2D air–liquid interface cultures, distinguished by apical–basal polarization and barrier integrity (Huang et al., 2020; Katsura et al., 2020; Lamers et al., 2021; Han et al., 2021), or as organoids in long-term feeder-free, chemically defined culture systems (Salahudeen et al., 2020). The expression of ACE2 and TMPRSS2 was equal to the adult stage, with more extensive expression of TMPRSS2 (Huang et al., 2020; Katsura et al., 2020; Salahudeen et al., 2020; Lamers et al., 2021; Han et al., 2021). Experiments showed that SARS-CoV-2 could infect and replicate in alveolar epithelial type II cells grown as either 3D organoids, 3D spheres, or 2D air–liquid interface cultures (Huang et al., 2020; Katsura et al., 2020; Salahudeen et al., 2020; Lamers et al., 2021; Han et al., 2021). All of these cell cultures managed to mirror viral infection and release of infectious virus predominantly from the apical side, following the expression of ACE2 protein. Furthermore, SARS-CoV-2 infection was examined in distal-lung basal cell-derived organoids (Salahudeen et al., 2020; Lamers et al., 2021). The SARS-CoV-2 infection led to pathological and apoptotic effects and a vigorous induction of host antiviral response genes, such as *IFN type I* and *type III*, IFN receptors and other interferon-stimulated genes (ISGs) referred to as type I and type III IFN responses, and NF-kB-mediated inflammatory signaling and chemokine signaling pathway (Han et al., 2021; Huang et al., 2020; Katsura et al., 2020; Lamers et al., 2021). There was an upregulation of apoptosis-related genes, while in infected cells certain functions of alveolar epithelial type II cells, such as surfactant gene expression, including DNA replication and cell cycle genes, were downregulated (Katsura et al., 2020). Even though primary cell cultures displayed potent IFN response (Lamers et al., 2020, 2021), alveolar epithelial type II cell organoids and PSC-derived 2D air–liquid interface cultures had a mild response (Han et al., 2021; Huang et al., 2020). Since cigarette smoke is displayed to enhance chances of a severe form of SARS-CoV-2 infection (Adams et al., 2020), the lungs were therefore examined in terms of androgens and cigarette consumption models (Purkayastha et al., 2020; Samuel et al., 2020). In a study of the primary human nonsmoker airway basal stem cell-derived air–liquid interface cultures, exposure to cigarette smoke prior to SARS-CoV-2 infection induced a 2- to 3-fold rise in viral load, increased the number of infected and apoptotic cells, hindered the normal airway basal stem cell repair response, and attenuated IFN response (Purkayastha et al., 2020). Besides lung organoids, intestinal organoids can serve as models for various infections such as coronavirus (which can be propagated *in vitro* so that the small intestine is an alternate infection route) (Zhou et al., 2017). To understand the tissue tropism of SARS-CoV-2, multiple research groups (Ardestani and Maedler, 2020; Yang et al., 2020; Han et al., 2021) resorted to organoid approaches. Previously, Monteil et al. (2020) demonstrated that SARS-CoV-2 could directly infect capillary organoids and kidney organoids, both derived from hiPSCs (Monteil et al., 2020). These observations may explain the spread of the virus through body and kidney function loss in severely ill individuals (Clevers, 2020).

## Conclusion

Rapid improvement of technology and growing urbanization require constant exposure to a plethora of various compounds, many of which are toxic. In such a manner, revealing the exact impact of pollutants on humans is imperative. Even though some studies elaborated precise mechanisms of pollutants *via* organoids including cytotoxic effects and decrease of cell viability, membrane integrity disruption, upregulation and downregulation of genes involved in cell growth differentiation and homeostasis, and increased levels of ROS, calcium oscillations, etc. (Yang et al., 2013; Kermanizadeh et al., 2014; Ferraz et al., 2016; Forsythe et al., 2018; Harris et al., 2018; Chen et al., 2019; Henson et al., 2019; Kim J. H. et al., 2020; Zeng et al., 2021; van Dijk et al., 2021, the contribution is a preprint; Winkler et al., 2021, the contribution is a preprint), other studies showed no adverse effects, or minimal cellular alterations (George et al., 2019; Di Cristo et al., 2020). Of great importance is consideration of specific features when it comes to organoids—lack of vasculature and immune system cells, cell-to-cell communication, ECM, and natural cell niche, even few, but are detrimental regarding the narrow portrayal of organoids’ reaction to pollutants. Also, the process of humans’ pollutant intake and exposure, even though mimicked to some extent, on the other hand, is not ideal. Despite of all these limitations, organoids will be a breakthrough in pollution research due to closer mirroring of human physiology, dodging of animal killing, comfortable manipulation with various compounds and dosage, excellent possibility to examine even the slightest changes in signal pathways, gene expression, and toxical effects of pollutants by different bioinformatics tools for analyzing omics such as *HiPathia* or others. Also, a wide range of applications regarding early development research, disease modeling (especially current SARS-CoV-2 virus infection), and testing of the vast majority of drug and toxicants overcome these limitations. In the aftermath, future directions should be aimed at overcoming limitations of 2D and 3D cell cultures, spreading and deepening the research with a much larger group of pollutants and cell types.

## Author Contributions

MS devised the idea. DM and DP wrote the manuscript. MP prepared the figure. MS and BLj provided the expert comments and edited the manuscript. MJ, SN, and NM wrote sections of the manuscript. All the authors contributed to the manuscript revision and read and approved the submitted version, which was completed by MS and BLj.

## Conflict of Interest

The authors declare that the research was conducted in the absence of any commercial or financial relationships that could be construed as a potential conflict of interest.

## Publisher’s Note

All claims expressed in this article are solely those of the authors and do not necessarily represent those of their affiliated organizations, or those of the publisher, the editors and the reviewers. Any product that may be evaluated in this article, or claim that may be made by its manufacturer, is not guaranteed or endorsed by the publisher.

## Abbreviations

ACE-2: angiotensin-converting enzyme 2
AEC: alveolar epithelial cell
AgNps: silver nanoparticles
AgO: silver oxide
AO: alveolar organoid
APOC3: apolipoprotein C3
ASCs: adult stem cells
AST: aspartate aminotransferase
AT2: alveolar type 2 progenitor cells
ATF4: activating transcription factor 4
BMP: bone morphogenetic protein
BRN2/POU3F2: POU class 3 homeobox 2
CASP3: caspase 3
CAT: catalase
CdTe: cadmium telluride
CeO2: cerium oxide
CF: cystic fibrosis
CLDN1: claudin 1
COPD: chronic obstructive pulmonary disease
COX1: cyclo-oxygenase 1
CRC: colorectal cancer
CTIP2/BCL11B: BAF chromatin remodeling complex subunit BCL11B
CuO: copper oxide
DAT: dopamine active transporter
DNAH5: dynein axonemal heavy chain 5
dPM2.5: diesel particulate matter 2.5
EAAC1: excitatory amino-acid carrier 1
EBs: embryoid bodies
ECM: extracellular matrix
ENps: engineered nanoparticles
ESCs: embryonic stem cells
Fbw7: F-box and WD repeat domain-containing 7
FGF: fibroblast growth factor
GABA receptor: gamma-aminobutyric acid receptor
GFAP: glial fibrillary acidic protein
GI: gastrointestinal
GLAST/SLC1A3: solute carrier family 1 member 3
GSH: glutathione
GSTA1 and GPX1: glutathione detox-related genes
hCG: human chorionic gonadotropin
HER: human epidermal growth factor receptor
hEROs: human embryonic stem cell derived retinal organoids
iHIOs: induced human intestinal organoids
iPSCs: induced pluripotent stem cells
ITER: international thermonuclear experimental reactor
Krt19: CK19 cytokeratin 19 protein
KRT5: keratin 5
LGR5: leucine-rich repeat-containing G-protein coupled receptor 5
LUHMES: Lund human mesencephalic cells
MLF1IP: centromere protein U
MPFs: microplastic fibers
MPs: microplastics
MT: microtissue
MWCNT: multiwalled carbon nanotubes
NADP: nicotinamide adenine dinucleotide phosphate
ND1: NADH dehydrogenase 1
NEF2L2: nuclear factor erythroid-derived 2-like 2)
Ngn3: neurogenin 3
NKX2.1: NK2 homeobox 1
NM: nanomaterial
NOX2: NADPH oxidase-2
NPHP1: nephrocystin 1
NPs: nanoplastics
Nps: nanoparticles
OrgGloms: organoid-derived glomeruli
PAHs: polycyclic aromatic hydrocarbons
PDGFRA: platelet-derived growth factor receptor alpha
PM2.5: particulate matter 2.5
PSCs: pluripotent stem cells
PSNPs: polystyrene nanoplastics
qRT-PCR: quantitative reverse transcription–polymerase chain reaction
RELN: reelin
RNA-seq: RNA sequencing
ROS: reactive oxygen species
SARS-CoV-2: severe acute respiratory syndrome coronavirus clade 2
SCGB1A1: secretoglobin family 1A member 1
SFTPA1: surfactant protein A1
SFTPC: surfactant protein C
SHH: Sonic hedgehog pathway
SOD1 and SOD2: superoxide dismutase family genes
TEER: transepithelial electrical resistance
TiO2: titanium dioxide
TMRPSS2: cofactor transmembrane protease serine 2
TSCs: trophoblast stem cells
TYMS: thymidylate synthetase
VEGF: vascular endothelial growth factor
VGAT: vesicular GABA transporter
VGLUT2: vesicular glutamate transporters
WES: whole-exome sequencing
WGS: whole-genome sequencing
W-Nps: tungsten nanoparticles
WNT: wingless-related integration site
ZnO: zinc oxide.
